# Commentary: Immunology's Coming of Age

**DOI:** 10.3389/fimmu.2019.02175

**Published:** 2019-09-12

**Authors:** Heinz Kohler, Anastas Dimitrov Pashov, Thomas Kieber-Emmons

**Affiliations:** ^1^Department of Microbiology and Immunology, University of Kentucky, Lexington, KY, United States; ^2^Stephan Angelov Institute of Microbiology, Bulgarian Academy of Sciences, Sofia, Bulgaria; ^3^Pathology, University of Arkansas for Medical Sciences, Little Rock, AR, United States

**Keywords:** antibody, idiotype, selection, natural antibody, therapeutic antibody

The recent review by Stefan Kaufmann on “Immunology's Coming of Age” is an elegant historical outline of the evolution of Immunology with focusing on a particular perspective of the history of Immunology, that is Nobel Laureate contributions to the discipline. Immunology is a difficult discipline to survey. Even the best attempts would ultimately focus on some selected aspects. As such, it invites comments aiming to complement the presented history in the context of Immunology coming of age. It is the aim of our Commentary to add important research in the field of immunology to demonstrate that it has become a self-containing discipline.

## Introduction

Immunology is a rich discipline with successes and failures, with various scholarly works describing its origins and history that lend to our current understanding of immunological principles (1, 2). Still another perspective has been presented recently by Stefan Kaufmann emphasizing notable contributions acknowledged by the awarding of a Nobel Prize to outstanding investigators (3). While touching on extremely important developments, important contextual elements need to be mentioned to complement the presented history as important contributions are not always recognized by a Nobel Prize.

## Discussion

### Antibody Recognition and Diversity

Saying that Immunology is an interdisciplinary science may no longer be entirely true since now it has also its own methods. The most prominent immunological paradigm is the concept of antibody. The specificity of antibodies is still an important question in immunology. Historically, the generation of diversity of antibodies was a hot discussed topic in the middle of the twentieth century initiated by the template hypothesis of Breinl and Haurowitz in 1930 (4), 10 years prior to Pauling's claim, cited in Kaufmann's review, that antibodies were made by folding newly synthesized nascent antibody polypeptide chains around the antigens, which serve as a template. Breinl and Haurowitz “thought that antibodies acquired their specificity for antigen by folding of the newly synthesized nascent polypeptide chain around the antigen” (5). The biochemical properties of antigen-antibody binding interactions were examined in more detail in the late 1930s by John Marrack (6). The biomolecule responsible for these actions was termed antitoxin, precipitin, and agglutinin. It was not known that all three substances were one entity. This was later demonstrated by Elvin A. Kabat showing the heterogeneity of antibodies through ultracentrifugation studies of horses' sera. Similarly, an equally important milestone in the understanding of Immunological recognition was the x-ray resolution of a Fab antibody fragment (7) not recognized in the review and the founding of the definition of antibody diversity and its biological significance by Kabat (8, 9). This work provided a transforming view of antibody diversity and the molecular basis for antigen recognition (10).

### Idiotype Hypothesis

Niels Jerne made several important contributions to Immunology. Niels Jerne's antibody selection theory is cited, but his more important contribution in the field of Immunobiology, the Idiotypic Network hypotheses, is not mentioned being essential for a historical record (11). He suggested that antibodies could be recognized as foreign, inducing other antibodies and thereby forming a network. Neglecting idiotype may be seen as more of a cultural aspect since it has not been accepted as a mainstream theory. Nevertheless, it has left a considerable imprint in immunological thinking. Recent reviews in Frontiers address the importance of the Idiotype concept in Immunology (12, 13). It might be argued that the Idiotypic Network hypothesis is the forerunner of present day ideas on the role antibodies plays in integrative Systems Immunology (14).

### Selection

Positive and negative selection (of both T and B cells) as well as the practical and theoretical aspects of intravenous immunoglobulins are important Immunology discoveries. The term “tolerance” was first coined by Ray Owen in reference to a physiological state he observed in dizygotic twin cattle (15) as noted in a review of the historical record of immunological tolerance (16). Just like antibodies, the elucidation of the T cell structure was monumental (17, 18). This facet provided the backdrop of monumental studies by Ellis Reinherz, Phillippa Marrack, John Kappler, and James Allison. Checkpoint inhibitors, which are driving Immmunotherapy, owe their existence to the understanding of how T cells in particular function.

### Natural Antibodies

Of no less importance is the regulatory and therapeutic potential of natural antibodies (19). Natural antibodies play an important role in the first line of defense and house keeping (20, 21). For a long period, natural antibodies were merely regarded as insignificant background of immunity. However, an early study in 1925 indicated that natural antibody in normal serum could neutralize bacteria (22).

### Therapeutic Antibodies

With the discovery of immortalizing antibodies by Kohler and Milstein (23) opened a new drug class to treat infections, auto-immunities and other diseases (24, 25). In parallel intravenous immunoglobulin (IVIg) emerged as standard therapy of immunoglobulin deviancies, auto-immune reactions and in homeostasis (26–28). These translationary aspects of Immunology deserve to be noticed.

## Conclusion

The History of Immunology began with Edward Jenner's discovery that vaccination protects against smallpox. Many scientists and discoveries have since lent to our understanding of how the immune system fights disease and sometimes causes disease as well to new classes of drugs. As we move closer to individualized medicine scenarios there will be a continuing need to understand and maybe redefine what came before and what will evolve in the discipline Immunology.

## Author Contributions

All authors listed have made a substantial, direct and intellectual contribution to the work, and approved it for publication.

### Conflict of Interest Statement

The authors declare that the research was conducted in the absence of any commercial or financial relationships that could be construed as a potential conflict of interest.
